# Transmedia storytelling usage of neural networks from a Universal Design for Learning perspective: A systematic review

**DOI:** 10.3389/fpsyg.2023.1119551

**Published:** 2023-03-28

**Authors:** Rafel Meyerhofer-Parra, Juan González-Martínez

**Affiliations:** Department of Pedagogy, University of Girona, Girona, Spain

**Keywords:** transmedia storytelling, universal design for learning, neuroscience, transmedia learning, teaching and learning (processes and methodology), active learning

## Abstract

The use of transmedia storytelling (TST) experiences is increasingly common in today's media ecology. Mediated by participatory culture, the role of the prosumer, and competency processes that connect with the reality of learners, the incorporation of storytelling motivates and deploys diverse didactic strategies. Considering the *engagement* generated by these strategies, and the need to promote literacies to provide competences to a plural society, a systematic review of the literature on transmedia storytelling experiences from the perspective of universal design for learning (UD-L) using PRISMA is carried out: *a priori*, we start from the idea that, if UD-L is based on the principles of educational neuroscience and TST, in turn, concretizes some of the guidelines of UD-L, TST can naturally result in a didactic approach that capitalizes on educational neuroscientific knowledge in a harmonious way with the digital context in which we live. The review analyzes a total of 50 articles from four databases: ERIC, Scopus, Web of Science, and Dialnet. The results show a low development of the checkpoints of the UD-L guides, and it is concluded that the most worked checkpoints are those closest to the definition of transmedia storytelling, followed by the foundational aspects of UD-L and, finally, aspects of access. Engagement is reflected in the experiences, but scaffolding is required to consolidate learning. In addition to this is the need to guarantee a true participatory culture, which requires the integration of more elements that incorporate accessibility into didactic strategies, offering learning possibilities for different styles and forms.

## 1. Introduction

This systematic literature review aims to identify the use of neural networks during transmedia storytelling experiences from a universal design for learning (UD-L) perspective.

The use of didactic strategies based on storytelling in teaching and learning processes is common, as it is a valuable resource for developing an affective–emotional link with the contents to be worked on Bruner (1986), Egan (1986). Storytelling has evolved from analog to digital and from digital to transmedia. Initially through a process of digitalization, then with the arrival of the internet and now with social media, new forms of consumption and participation are emerging, where users can assume the role of prosumer: not only consuming resources but also participating fully in the creation and deployment of them (Toffler, 1980). In order to enable full participation in this new media ecosystem, there is an emerging educational need to integrate these competences and make them literate in formal educational processes, reinforcing—and in some cases laying the foundations for—the development carried out in informal and non-formal learning contexts, and thus connecting their learning with their reality outside school (Jenkins, 2006; Jenkins et al., 2009; Scolari, 2013, 2018; Jenkins and Ito, 2015; Faria-Ferreira et al., 2021).

In addition to all of this is a great opportunity: working in a transmedia way opens up many possibilities for personalization and adaptation of the learning process according to the educational needs required (Pence, 2012; Rodrigues and Bidarra, 2014, 2015; Gambarato and Dabagian, 2016; Sánchez-Caballé and González-Martínez, 2022). Moreover, in such possibilities, the UD-L paradigm finds a methodology that fits very well with the transmedia approach almost “naturally,” without the teacher having to force excessively to seek convergence with the principles of universality (González-Martínez, 2022): Elements that transmedia narratives offer through participatory culture, a prosumer role, and multiple media through which a story unfolds (Pineda Acero et al., 2018), all at the learner's choice to a large extent and with remarkable flexibility (González-Martínez, 2022). In that sense, UD-L is an approach that allows “to maximize learning at individual paces,” offering different entry and exit points to the learning process, offering a wide spectrum of modes of representation, consumption, and strategies for learning, so that each learner can roam within those possibilities and not only learn according to his or her uniqueness but also his or her interests. Moreover, it has, at its core, the attempt to capitalize on the knowledge generated by both experience and research and, as mentioned earlier, advances in educational neuroscience, which explain how we learn (Rose et al., 2006; Robinson and Wizer, 2016; Yuan et al., 2017). At its core, UD-L proposes to be guided, in the didactic design of learning experiences, by three principles, which are broken down into different (more concrete) lower order rules or recommendations: providing multiple forms of representation (principle 1), action and expression (principle 2), and engagement (principle 3). In addition to this, of course, in order to seek the maximum use of the different neural networks and their different weights in the different moments and elements of learning (Rapp, 2014; Alba Pastor, 2016; Castro and Rodríguez, 2017). We would say, then, chaining syllogisms, that transmedia storytelling is an intuitive way of aligning with the principles of UD-L and, therefore, it is sensible to think that it can also be a didactic way of harmonizing with the advances in educational neuroscience (Savia, 2015, 2018).

In summary, both transmedia storytelling and UD-L, insofar as they offer a great multimodality of possibilities in educational practice, are very interesting from a neuroscientific perspective, although we know little about these aspects in practice: What are the neural networks they focus on through their educational proposals? Do they work on affective, strategic, and recognition networks? Within each network: What elements do they develop?

It is well known that transmedia storytelling generates strong engagement, but there are authors who go a step further and link transmedia strategies to Zull's cone of emotion and Kolb's learning cycle (Kalogeras, 2013). Although, what this engagement entails has not been studied in such detail, since a lot of transmedia storytelling experiences lean toward sharing the practice rather than a reflection toward the methodological benefits, let alone what strategies and forms of representation they use if it is analyzed from a UD-L perspective.

In view of the aforementioned points, the current systematic review of the literature raises the following research questions, which can be observed in Table 1.

**Table 1:** Research questions of the study.

	**Research questions**
1	How are recognition networks used in Transmedia Storytelling experiences based on UD-L principles?
2	How are strategic networks used in Transmedia Storytelling experiences based on UD-L principles?
3	How are affective networks used in Transmedia Storytelling experiences based on UD-L principles?

## 2. Methods

In view of the initial research questions established, it is considered that the systematic literature review (SLR) using the PRISMA guidelines is a feasible method that will enable to locate and analyze the most relevant documents regarding the research questions while offering clear, useful, and replicable access to the research process (Okoli and Schabram, 2010; Urrútia and Bonfill, 2010). An SLR is “a systematic, explicit, comprehensive, and reproducible method for identifying, evaluating, and synthesizing the existing body of completed and recorded work produced by researchers, scholars, and practitioners” (Okoli, 2015, p. 880).

### 2.1. Search strategy

The search for theoretical essays and studies related to transmedia storytelling was carried out in four electronic databases with the terms of “transmedia” AND “education”. The search choice was “transmedia” and not “*transmedia storytelling*,” to widen up the initial identification and screen it manually rather than with the searching tools themselves, guaranteeing that only documents clearly related to transmedia storytelling experiences. In addition, it helped to use the same keywords in all the databases, which could have been an issue while using non-English databases. Since one of the databases is in Spanish, the decision was relevant to recollect articles that translated “storytelling” to “narratives”.

### 2.2. Information sources

The databases chosen were Web of Science for its quality and inclusion of all types of publications; Scopus, for its recognized content of quality scientific articles; Educational Resources Information Center (ERIC), for being a repository focused on research in the field of education; and Dialnet, a database with documents published in Spanish, since large numbers of publications in the field of transmedia storytelling are in this language.

### 2.3. Selection process

The selection criteria used were based on van Eerd et al. (2010) and Mahood et al. (2013) and were open to all types of published publications (book chapters, conferences, and PhD Theses.) but must define or include implicitly or explicitly what they understand as transmedia storytelling. Documents not in English or Romanic languages were excluded.

The document relevance review was first based on the review of the abstract or, if necessary, the full document. Pairs of reviewers blindly voted to take into consideration if the document included a definition of transmedia storytelling or, alternatively, that they use the concept of transmedia storytelling and describe, more or less explicitly, what they are referring to while using the concept. Reviewers were guided initially by definitions in the literature such as Jenkins (2006) and Phillips (2012) but also by the identification of coexistent concepts such as remixing, participatory culture, and prosumer (González-Martínez et al., 2019).

Once the first round of blind votes was conducted, they were merged. In case of agreement, the document was included or excluded from retrieval. If there was not an initial consensus between the two researchers, a third peer blindly voted for three different options: inclusion, exclusion, or doubt, leading the latter to a process of discussion of how to proceed regarding that article. Only documents with enough detail on content were judged according to the quality criteria, which can be seen in Table 2.

**Table 2:** Quality criteria questions and scores.

	**Question**	**Score**
1	Was the purpose of the paper stated clearly	2
2	Was the rationale for implementing a transmedia experience intervention described	2
3	Were the steps of the intervention clearly outlined	4
4	Were the media/s used in the experience clearly identified	2
5	Were the principles of UD-L integrated in the experience	2
6	Does the document describe the impact of the transmedia experience	2
7	Does the user participate in the unfolding of the story?	2

Based on van Eerd et al. (2010) and Mahood et al. (2013).

Partial scores (half value) were possible for questions 1–5. A maximum of 16 points can be achieved. Data extraction was performed with those documents receiving the quality criteria score of 10 or more (out of 16). In addition, it was initially planned that if one of the reviewers identified a document as rich content referring to its transmedia storytelling experience, or its consideration toward UD-L, it would be retained for data extraction even if the quality score was lower than 10. In the end, this measure was planned but not used, since all the documents identified as relevant by the reviewers had a high-quality score.

**Figure 1:**
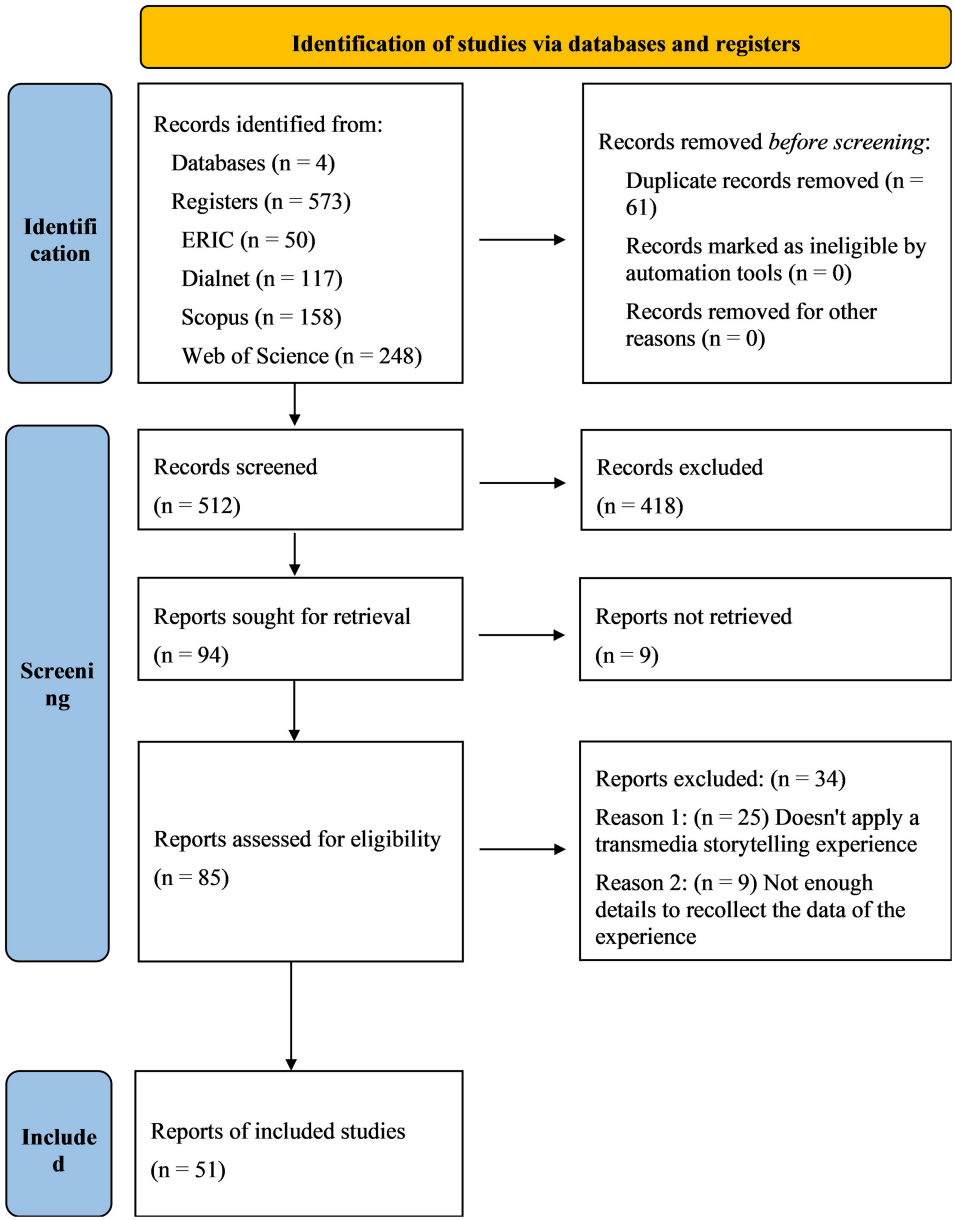
Identification of studies via databases and registers using PRISMA 2020 (Page et al., 2021). For more information, visit: http://www.prisma-statement.org/.

A more detailed explanation of the procedure, expanding the information on the identification and screening process, can be observed in Figure 1, and a synthesis of the included studies is shown in Table 3.

**Table 3:** List of included studies.

**Num**.	**References**	**Doc. type**	**Educational context/ level**	**Geograph ical context**
[1]	Diéguez (2014)	Paper	Master and undergraduate	Hungary and USA
[2]	Jover et al. (2015)	Paper	Secondary	Spain and Chile
[3]	Peña-Acuña (2021)	Paper	Primary and Secondary	Spain
[4]	Faria-Ferreira et al. (2021)	Paper	Primary	Portugal
[5]	Vásquez Arias and Montoya Bermúdez (2016)	Paper	Secondary	Colombia
[6]	Rovira-Collado et al. (2016)	Paper	University—Master	Spain
[7]	Charria Castaño (2017)	Paper	Secondary	Colombia
[8]	Molas Castells and Rodríguez Illera (2018)	Paper	Secondary	Spain
[9]	Arrausi Valdezate and Cerro Villanueva (2017)	Paper	Non formal—Linked to business	Spain
[10]	Gutiérrez Pequeño et al. (2017)	Paper	University—undergraduate	Spain
[11]	Molas Castells and Rodríguez Illera (2018)	Paper	Secondary	Spain
[12]	Gómez-Trigueros et al. (2018)	Paper	University—master	Spain
[13]	de la Puente (2018)	Paper	University—graduate students	Argentina
[14]	Scolari et al. (2019)	Paper	Secondary	Spain
[15]	Amador-Baquiro (2018)	Paper	Primary and secondary	Colombia
[16]	Albarello and Mihal (2018)	Paper	Primary and secondary	Argentina
[17]	Alonso and Murgia (2018)	Paper	Secondary	Argentina
[18]	Tomšič Amon (2019)	Book chapter	University—undergraduate	Slovenia
[19]	Tomšič Amon (2020)	Paper	University—undergraduate	Slovenia
[20]	Gambarato and Dabagian (2016)	Paper	Not defined	Canada and USA
[21]	Hovious et al. (2021)	Paper	Secondary	USA
[22]	Reid and Gilardi (2016)	Book chapter	University—undergraduate	Japan
[23]	Scolari et al. (2020)	Paper	Secondary	Spain
[24]	McCarthy et al. (2018)	Paper	Pre-primary	USA
[25]	Stansell et al. (2015)	Paper	Primary and secondary	USA
[26]	Benedict et al. (2013)	Paper	University—undergraduate	USA
[27]	Rodríguez-Illera and Molas-Castells (2014)	Paper	Secondary	Spain
[28]	Paulsen and Andrews (2014)	Paper	Pre-primary	USA
[29]	Kalogeras (2013)	Paper	University	UK
[30]	Fleming (2013)	Paper	Secondary	USA
[31]	Myers (2020)	Paper	Non formal context	Australia
[32]	Wiklund-Engblom et al. (2014)	Paper	University—undergraduate	Finland
[33]	Coles and Bryer (2018)	Paper	University—undergraduate	UK
[34]	Lachman et al. (2010)	Paper	Secondary	Canada
[35]	Wiklund-Engblom et al. (2013)	Conference paper	University—undergraduate	Finland
[36]	Ellis et al. (2018)	Paper	Secondary	USA
[37]	Berezina (2020)	Paper	University—undergraduate	Malaysia
[38]	Perry (2020)	Paper	University—undergraduate	Malaysia
[39]	Arkhangelsky et al. (2021)	Paper	Primary	Russia
[40]	Rodrigues and Bidarra (2014)	Paper	Secondary	Portugal
[41]	Raybourn (2014)	Paper	Non formal—Army	USA
[42]	Cronin (2016)	Paper	University	USA
[43]	Heilemann et al. (2018)	Paper	Non formal —medical use for women with depression	USA
[43]	Anguita Martínez et al. (2018)	Paper	University—undergraduate	Spain
[44]	Fernández Díaz et al. (2019)	Paper	University—undergraduate	Spain
[45]	Rosenfeld et al. (2019)	Paper	Preschool	USA
[46]	Djonov et al. (2021)	Paper	Pre-primary	n.d.
[47]	Marín-García and Gómez (2014)	Paper	University—undergraduate	Spain
[48]	Rodrigues and Bidarra (2019)	Conference paper	Secondary	Portugal
[49]	Peralta García and Ouariachi Peralta (2015)	Paper	Post secondary education	Spain
[50]	Bárcenas López et al. (2018)	Paper	Upper secondary	Mexico
[51]	Herrero (2019)	Book chapter	Secondary	UK

### 2.4. Data collection process

In order to collect information through which to answer the research questions, we use the UD-L guidelines presented by CAST (2018), in which there are three levels under which to collect transmedia experiences, their use of UD-L principles, and how they connect to networks.

On the first level, there are three networks (*recognition, action and expression*, and *engagement*). On the second level, the nine UD-L guidelines related to the three networks (*recognition*: perception, language, and symbols; *action and expression*: comprehension, physical action, expression and communication, and executive functions; and *engagement*: recruiting interest, sustaining effort and persistence, and self-regulation). The neural networks and the guides also have proposed checkpoints, which can be seen in detail in Table 4.

**Table 4:** Networks, guidelines, and checkpoints collected during the screening process of the systematic review according to explicit (E), implicit (I), and not developed (ND).

**Network**	**Guidelines**	**Checkpoints**	**E**	**I**	**ND**
Representation	G1. Perception	C1.1. Offer ways of customizing the display of information	14	4	33
		C1.2. Offer alternatives for auditory information	4	2	45
		C1.3. Offer alternatives for visual information	3	2	46
	G2. Language and Symbols	C2.1. Clarify vocabulary and symbols	1	1	49
		C2.2. Clarify syntax and structure	1	0	50
		C2.3. Support decoding of text, mathematical notation, and symbols	0	1	50
		C2.4. Promote understanding across languages	1	10	40
		C2.5. Illustrate through multiple media	7	10	34
	G3. Comprehension	C3.1. Activate or supply background knowledge	13	21	17
		C3.2. Highlight patterns, critical features, big ideas, and relationships	18	8	25
		C3.3. Guide information processing and visualization	24	6	21
		C3.4. Maximize transfer and generalization	7	14	30
Action and expression	G4. Physical Action	C4.1. Vary the methods for response and navigation	29	14	8
		C4.2. Optimize access to tools and assistive technologies	8	14	29
	G5. Expression and Communication	C5.1. Use multiple media for communication	36	6	9
		C5.2. Use multiple tools for construction and composition	38	7	6
		C5.3. Build fluencies with graduated levels of support for practice and performance	7	7	37
	G6. Executive Functions	C6.1. Guide appropriate goal setting	15	22	14
		C6.2. Support planning and strategy development	16	14	21
		C6.3. Facilitate managing information and resources	13	11	27
		C6.4. Enhance capacity for monitoring progress	8	8	35
Engagement	G7. Recruiting interest	C7.1. Optimize individual choice and autonomy	12	19	20
		C7.2. Optimize relevance, value, and authenticity	10	23	18
		C7.3. Minimize threats and distractions	0	5	46
	G8. Sustaining effort and persistence	C8.1. Heighten salience of goals and objectives	15	8	28
		C8.2. Vary demands and resources to optimize challenge	25	11	15
		C8.3. Foster collaboration and community	33	5	13
		C8.4. Increase mastery-oriented feedback	4	11	36
	G9. Self-regulation	C9.1. Promote expectations and beliefs that optimize motivation	9	18	24
		C9.2. Facilitate personal coping skills and strategies	8	20	23
		C9.3. Develop self-assessment and reflection	14	16	21

Retrieved from CAST (2018).

To collect the results of the experiences, it was decided to use three categories: explicit, implicit, and not developed. In this way, it will be possible to perceive the multiplicity of scenarios within the experiences, as well as the combination of different elements within the UD-L paradigm, understanding it as an opportunity to see which elements to incorporate within the experiences. An experience does not necessarily have to develop all the elements: a better experience is not one that includes more checkpoints within the guidelines, but tracking its use can help to see future perspectives within the application of UD-L in the educational field and, specifically, in the application of transmedia storytelling experiences.

In addition, it was decided to use another categorical system offered by CAST (2018) to contrast the information. This is why it is then analyzed according to *representation, action and expression, and engagement* and subsequently analyzed using access, which encompasses G1, G4, and G7; build, which contains G2, G5, and G8; and internalize, which has the last three: G3, G6, and G9.

## 3. Results

### 3.1. Action and expression, engagement, and representation

**Figure 2:**
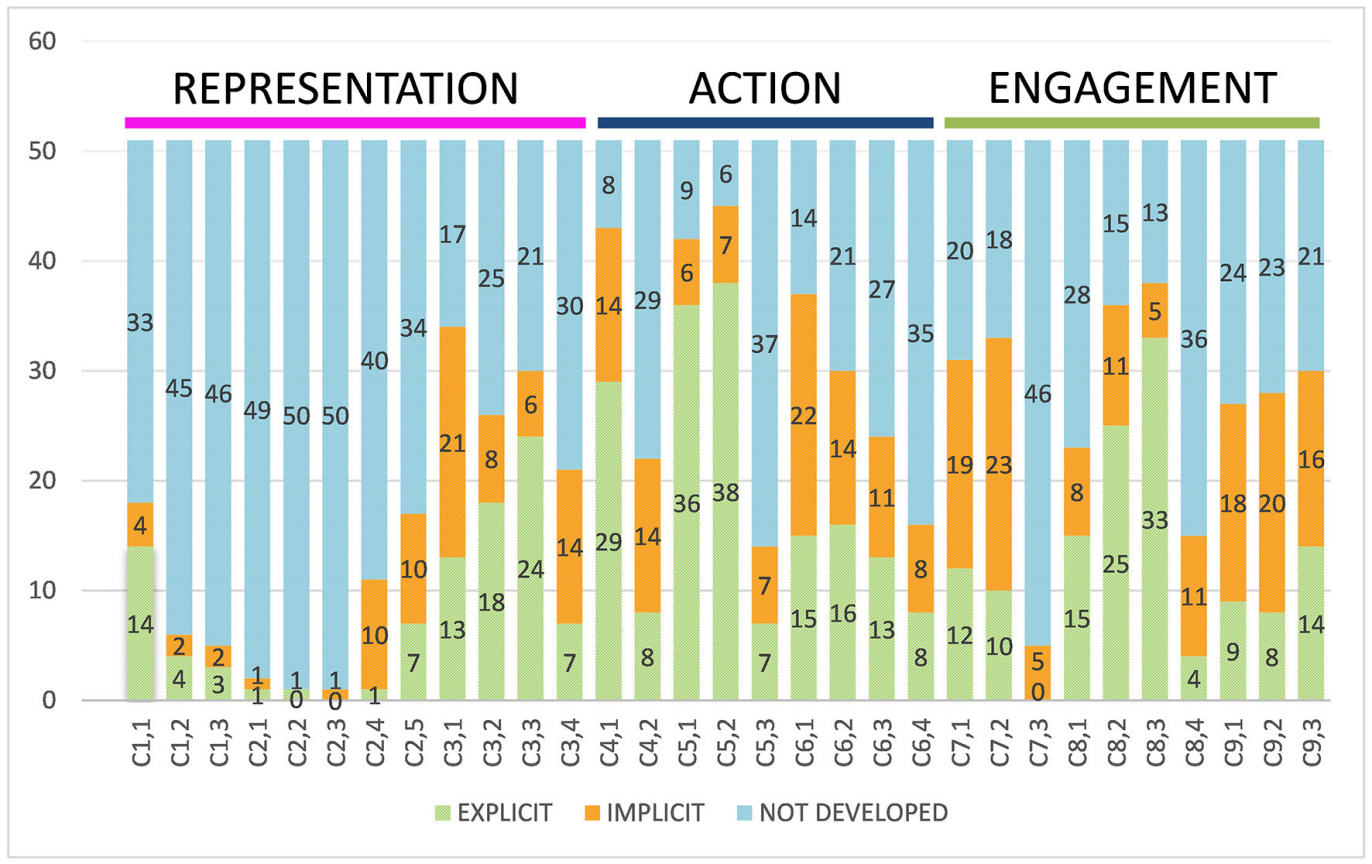
Explicit, implicit, and not developed uses of the checkpoints of the three networks.

As can be seen in Figure 2, with reference to the checkpoints sorted according to the categories of action and expression, engagement, and representation, there is a large divergence between the categories.

#### 3.1.1. Representation

Focusing initially on the representation category, it can be observed that in guideline 1, which includes alternative ways of providing information by visual or auditory means, practically no use is found in the experiences collected. Among the few experiences that include it are Faria-Ferreira et al. (2021), which provide alternative ways of reading, such as film fragments that correspond to the chapter, and an e-book, to change the sequence of the story without changing its meaning. Another case that seeks to offer alternatives is Scolari et al. (2019), with hypertextual adaptation by students to facilitate each other's reading, and then exploring adaptations of the work into graphic novels, narrative illustrations, and other graphic works. In fact, literally one of the activities proposed is the adaptation of graphic media, which in addition to offering alternatives to visual information, offers the opportunity to appropriate the text, generating a more immersive educational experience and thereby increasing intrinsic motivation with the learning process. In Albarello and Mihal (2018), it is also possible to identify how crossmedia elements are used to offer different access points to the information presented. In reference to checkpoint C.1.3, few alternatives for visual information are detected, and the podcast format is little identified as an option deployed to facilitate visual to auditory information.

Similarly, in guideline 2, a minority use is identified in the experiences, but both C2.4, which refers to *understanding across languages*, and C2.5, which refers to *illustrate through multiple media*, are used significantly more, being found to be developed in 17 and 21 experiences, while the other checkpoints were used between one and two times.

Within C2.3, *support decoding of text, mathematical notation, and symbols*, Tomšič Amon (2020), and his deployment of an experience that asks students to hybridize artistic and mathematical knowledge, so that they have to precisely identify the mathematical figures in a series of images, stands out. They find this particularly challenging to combine and are given both support to do this, and different levels of difficulty to work through. In C2.4, *promote understanding across languages*, and in C2.5, *illustrate through multiple media*, the cases identified are higher. Most of the cases identified in C2.4 are part of transmedia experiences linked to second language learning, and that is why the checkpoint is often more or less explicitly identified. Regarding C2.5, some studies make it more explicit than others: For instance, some initially present a map, graphic, or system to present what and how to work on it (Vásquez Arias and Montoya Bermúdez, 2016; Alonso and Murgia, 2018; de la Puente, 2018; Ellis et al., 2018; Pineda Acero et al., 2018; Rodrigues and Bidarra, 2019; Perry, 2020), which can also be related to C3.3 *Guide information processing and visualization*. The last guideline within representation is comprehension, and in it we can identify a higher use, finding it in more than half of the experiences analyzed. These are checkpoints that refer to prior knowledge (C3.1), patterns and relationships (C3.2); processing and visualizing information (C3.3); and its transfer (C3.4) and are considered by the experiences much more than the other guidelines within the same category.

During the screening process, it has been identified that transmedia storytelling often wants to connect informal and formal educational contexts, which connects with C3.1, C3.2, and C3.4, (Marín-García and Gómez, 2014; Gutiérrez Pequeño et al., 2017; González-Martínez, 2022), by choosing competences acquired outside and integrating them in the classroom (Albarello and Mihal, 2018; Alonso and Murgia, 2018; Scolari et al., 2020), or simply because the narrative moves the reflection to the present—students in the experiences often have to incorporate their prior knowledge for the development of the activity (Rodríguez-Illera and Molas-Castells, 2014; Albarello and Mihal, 2018; Alonso and Murgia, 2018; Gómez-Trigueros et al., 2018; Molas Castells and Rodríguez Illera, 2018; Scolari et al., 2019; González-Martínez, 2022). In addition, there is a latent need for *scaffolding*, which is not always present and assumes competences and literacies that they do not necessarily need to have (Kalogeras, 2013; McCarthy et al., 2018).

#### 3.1.2. Action and expression

Moving on to the *Action category*, the most represented checkpoints in the documents included in the SLR are identified. Starting with guideline 4, we find with 43 (29 explicit and 14 implicit) *vary the methods for response and navigation* and with 22 (8 explicit and 14 implicit) the C4.2 *Optimize access to tools and assistive technologies*. There is a very important difference between the two elements of the category, and it is especially noteworthy that most of the transmedia storytelling experiences. Both highlight their implicit use, often behind the alternatives that can be offered within digital tools, but especially in the case of C4.1, the fact that the learner can become a prosumer and the center of their learning process, where they can choose the media for their learning process, makes it easier for them to find alternatives within the requirements that suit their particularities.

Entering guideline 5, there is a disparity in its use: C5.1 and C5.2 are widely used, with C5.1 being one of the strong points in terms of composition in multiple media: illustrations, storyboards, films, video games, augmented reality games, simulations, chats, blogs, and comics, and in the case of C5.2 its use is also outstanding, but above all for the possibility of using hypertexts, concept maps, and many web resources. On the contrary, C5.3 is related to establishing graduated levels of support for practice and development, practically not identified in the experiences, with a very important contrast from 9 and 6 not used in the first two, to 37 not used for C5.3. In reference to C5.3, although an effort is made to try to give examples of good practices of transmedia narratives, or a certain background prior to the development of the activity, there is often no scaffolding either of competence or of the activity.

In the last guideline within action and expression, G6. *For executive functions, there* is a great heterogeneity of explicit, implicit, and not developed uses. Altogether, these items identify an inherent limitation in the research process: when they describe or talk about the experience, they do so as an object of study about which research questions were asked, and not always as a mere description of the activity carried out in the educational context. Therefore, some elements such as the setting of objectives, the setting of strategies, and the deployment of these and the other checkpoints in guideline 6 may be under-represented.

#### 3.1.3. Engagement

With regard to *guideline 7, recruiting interest*, C7.1 and C.7.2 are mostly used, with high values referring to individual decision and relevance or authenticity, while C7.3 refers to factors that will minimize distractions and are not developed in 46 of the 50 articles in the systematic review. Again, given that C7.1 and C7.2 are closely linked to the possibility of prosumption and an active role in the learner's unfolding of the story, it is common for them to be worked out, although it is cast in more open, and not so constantly guided transmedia experiences. In some cases (Albarello and Mihal, 2018; McCarthy et al., 2018; Rodrigues and Bidarra, 2019; Scolari et al., 2019), a very wide range of possibilities is identified. Even by proposing itineraries with compulsory and optional options, so that students can deploy what most motivates them and promote their autonomy both as a group and as individuals.

In guideline 8, there is a disparity of explicit, implicit, and not developed uses. In C8.2 and C8.3, a majority of the use of checkpoints can be seen (36 and 38), while in C8.1 it is moderate (23) and in C8.4 it is very low (15). In fact, it makes sense that both C8.2 and C8.3 are in line with each other, given that they are about varying demands and resources in order to optimize the challenge and to collect collaboration and community: two items closely linked to Jenkins' (2006) ideas on participatory culture and one of the phenomena he describes as paradigmatic in the current media ecology. In contrast, C8.1 and C8.4 refer more to the assessment process, which can often fall more on the teacher.

To conclude with the analysis of checkpoints, the last of the engagement network guidelines is self-regulation. In this guideline, there is a moderate use of checkpoints, but they are mostly detected implicitly and identified in the discourse, rather than explicitly (C9.1. 9–18; C9.2. 8–20; C9.3. 14–16). Often the elements of *self-regulation* were identified during the review process through the description of what tasks were given to learners, rather than through a process of making them explicit or inviting them to perform those tasks. One of the elements claimed in the reflections is the aspect of motivation (Albarello and Mihal, 2018; Heilemann et al., 2018; McCarthy et al., 2018; Molas Castells and Rodríguez Illera, 2018; Scolari et al., 2019; Perry, 2020; Hovious et al., 2021).

### 3.2. Categories from a general perspective

**Figure 3:**
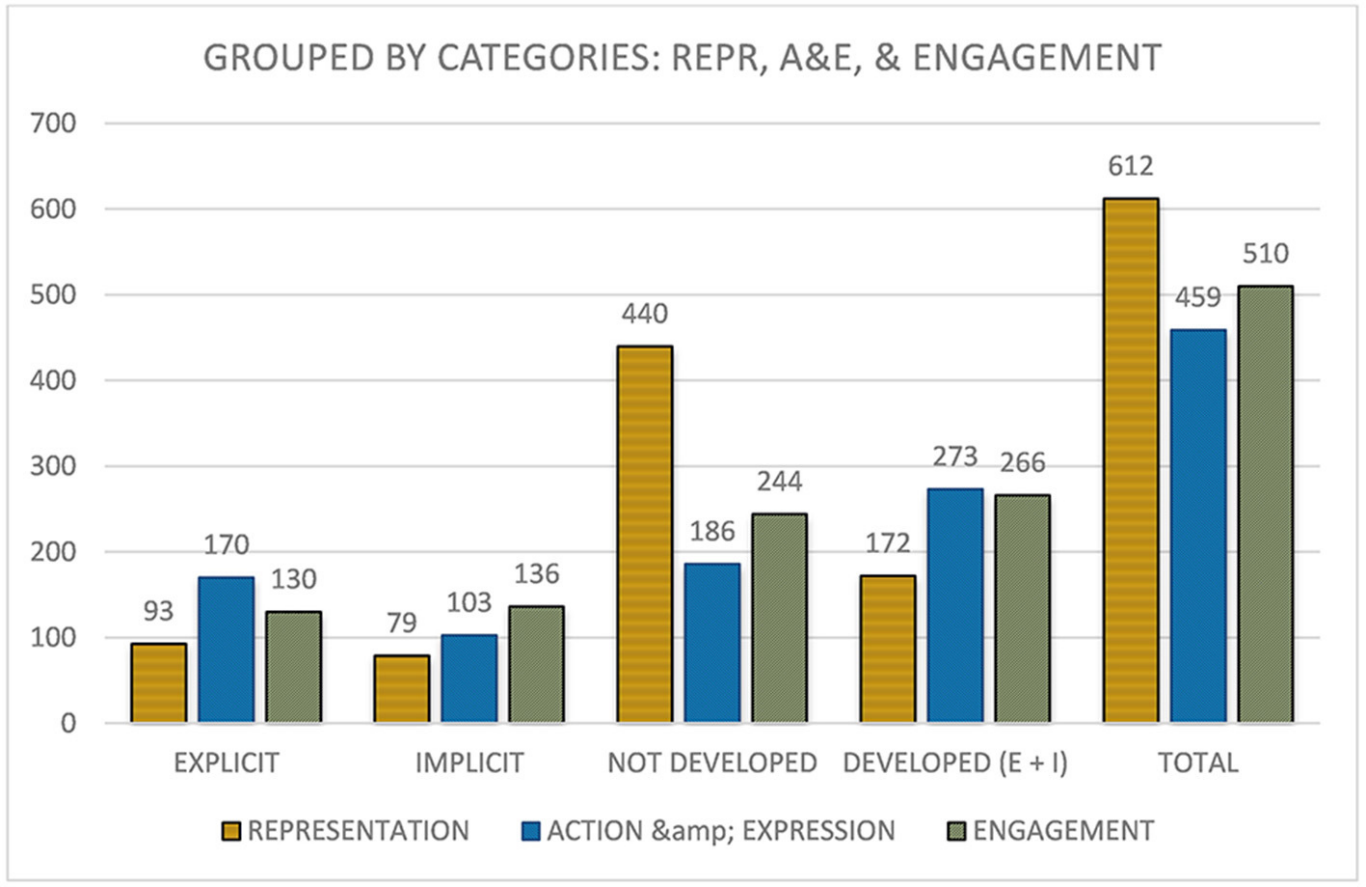
Identified uses of checkpoints grouped by the three networks.

Moving toward the analysis of the guidelines in a more general perspective, and taking into consideration the aggregate data of the aforementioned checkpoints seen in Figure 3, we can raise different considerations.

First, as can be seen in Figure 3, the representation network has the most items and, therefore, a higher total aggregate (612). It is followed by *engagement* (510) and action and expression (459). Although, it is surprising how *representation* is the network with the highest number of possible points that has precisely the fewest, with 15.20% of explicit uses, 12.91% of implicit uses, and 71.89% of not developed uses. This is a very high percentage and contrasts with the other networks, which have 47.84 (*engagement*) and 40.53% (*action and expression*) of not developed.

Moving on to the *engagement* network, the figures for this network are quite different: Explicit and implicit use are quite similar (25.49 and 26.67%, respectively), totaling 52.16%, just over half of which have been explicitly identified, and only a quarter of which have been explicitly identified. Finally, with regard to the action and expression network, there is a greater explicit use of checkpoints (37.03%) and an implicit use of 22.44%, which in aggregate is the highest use identified among the three networks (59.48%).

Taking into consideration a large amount of not developed data, it is decided to expose the results also according to the *access, build, and internalize* categories.

### 3.3. Access, build, and internalize

**Figure 4:**
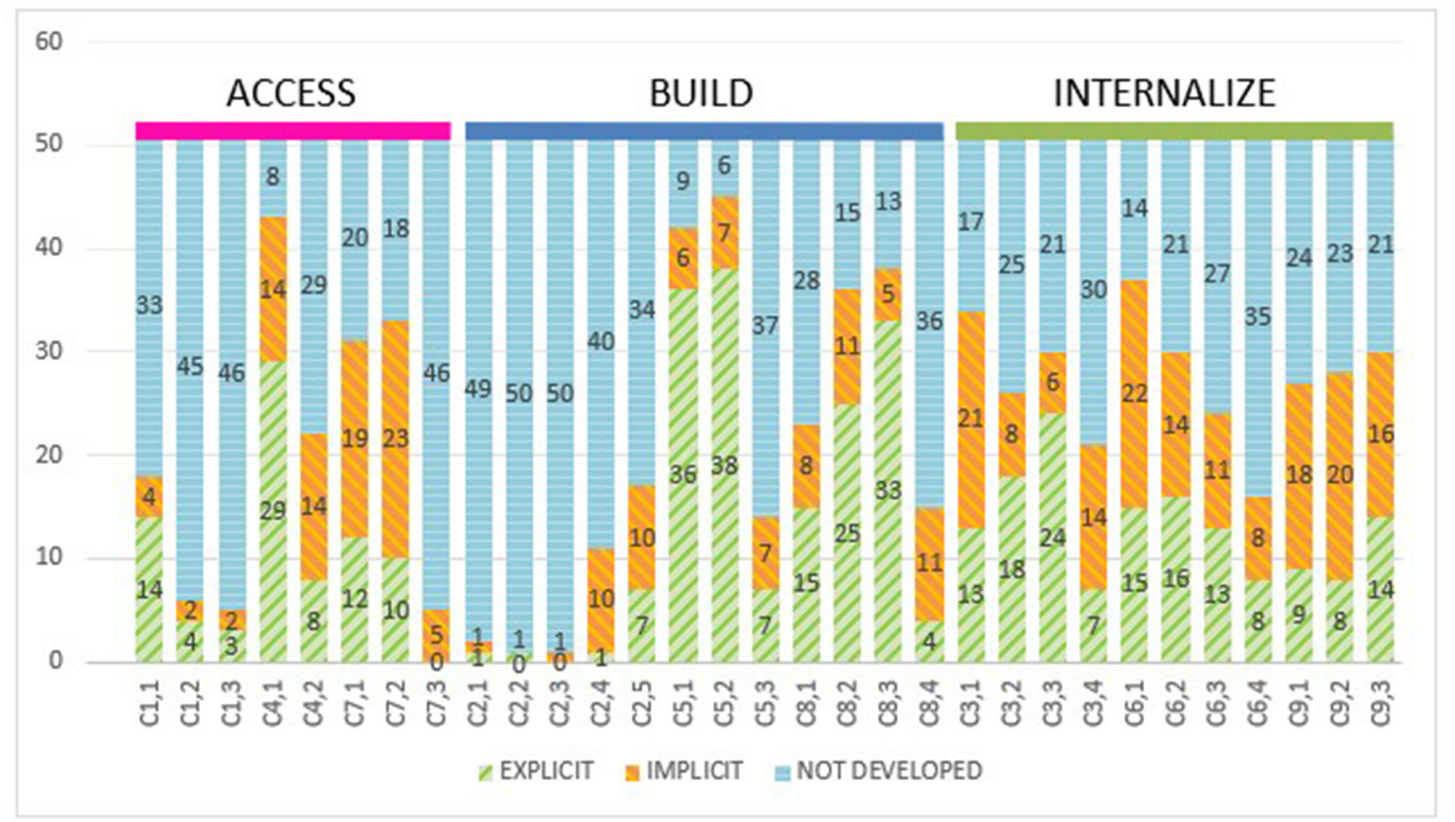
Explicit, implicit, and not developed uses sorted by *access, build, and internalize* categories.

As can be seen in Figure 4, access is made up of guidelines 1, 4, and 7. Looking at the explicit, implicit, and not developed uses through the access perspective, a great divergence in the data can be observed. While the checkpoints of guideline 1 have very low values, those of guidelines 4 and 7 are among the highest, which allows us to identify that the experiences may not develop some of the items in the access perspective, but do take access into consideration within their proposals, although in a way that is closer to the engagement perspective (G7) and action and expression (G4), and not to the representation perspective (G1).

As far as build is concerned, a similar situation is identified: There is a very low use of guideline 2, which corresponds to representation, but a high use of the checkpoints corresponding to action and expression (G5) and engagement (G7).

Finally, analyzing from the perspective of the internalized category, balanced use of the checkpoints corresponding to the representation (G3), action and expression (G6), and engagement (G9) is identified.

If these data are observed and grouped by category, significant differences in the weighting of the categories become apparent.

**Figure 5:**
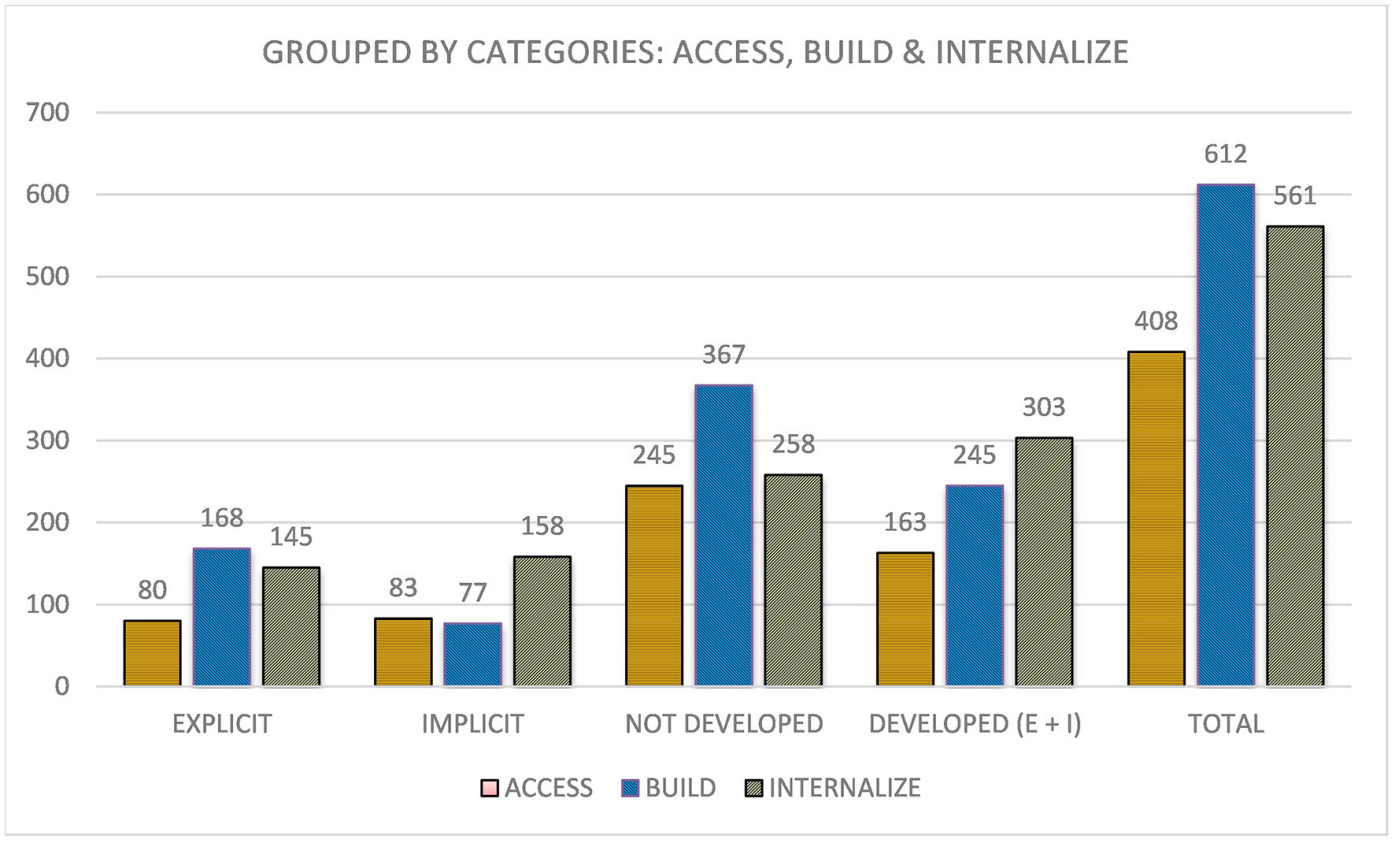
Identified uses of checkpoints grouped by access, build, and internalize categories.

As can be seen in Figure 5, first, the access category has a much smaller representation (408) compared with the build (612) and internalize (561). Going into the uses, access has a very similar explicit and implicit use (80 and 83), corresponding to 39.95% (19.61 and 20.34%, respectively) of the total, in contrast to the 60.05% of not developed (see Table 5).

**Table 5:** Percentage of explicit, implicit, and not developed use.

	**Explicit (%)**	**Implicit (%)**	**Not developed (%)**
Access	19.61	20.34	60.05
Build	27.45	12.58	59.97
Internalize	25.85	28.16	45.99
Engagement	25.49	26.67	47.84
Representation	15.20	12.91	71.89
Action and expression	37.03	22.44	40.53

Second, in the build category, explicit usage (27.45%) is significantly higher than implicit usage (12.58%), which in aggregate is 40.03%, and both the build and access categories have very similar developed—not developed data.

Finally, the *internalize category* shows very similar explicit and implicit use (25.85 and 28.16%), which are substantially higher than in the other categories, totaling 54.01%. Considering that this is the highest figure within the three grouped categories, it helps to visualize that a large number of checkpoints are not worked from the three categories.

### 3.4. Comparison of the categories

Note that when calculating the development in terms of the triad access, build, and internalize, the percentages are quite different from those observed in terms of *engagement, representation, and action and expression*.

**Figure 6:**
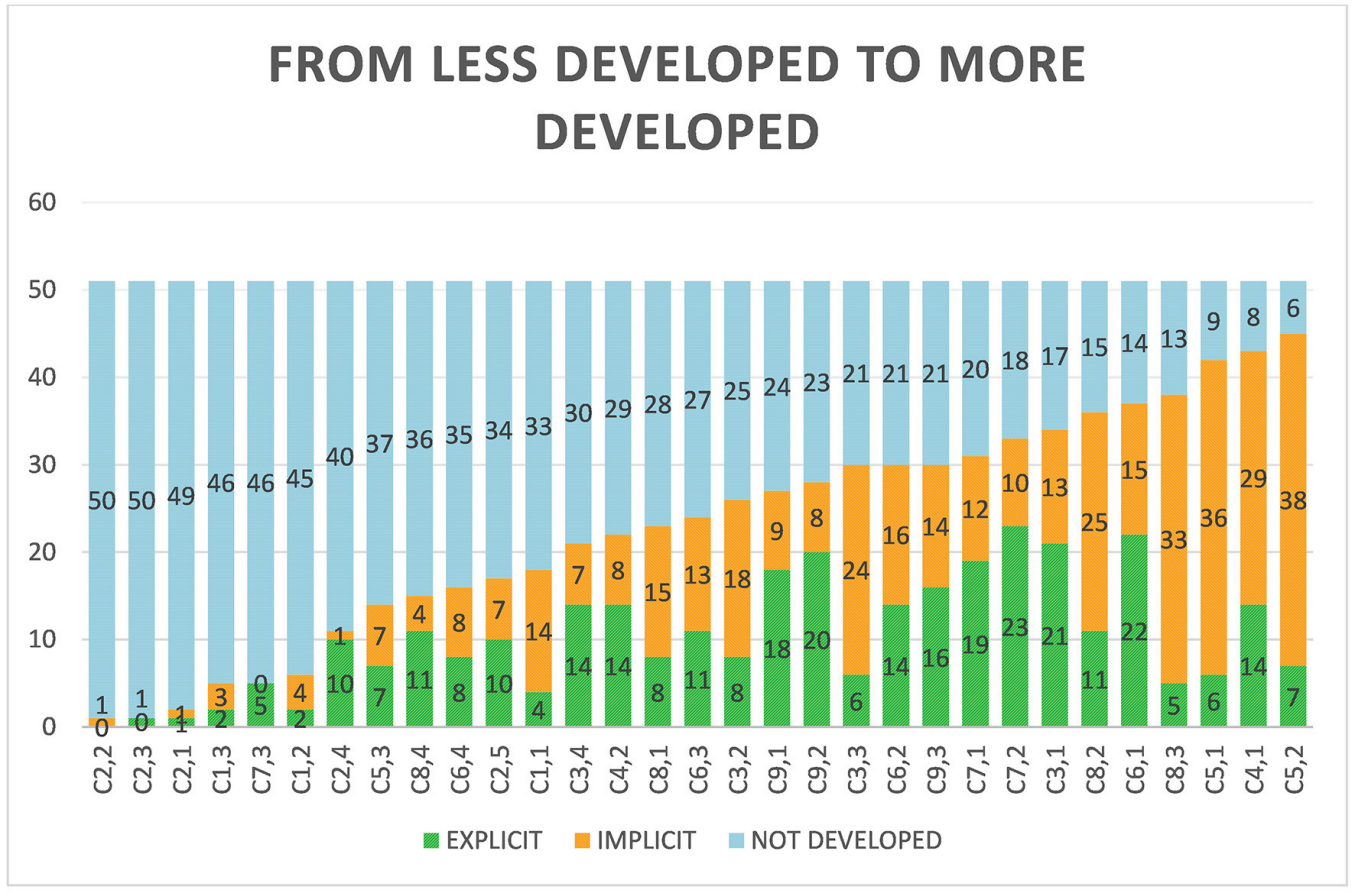
Implicit, explicit, and not developed uses. Sorted from the most to the least not developed.

Finally, focusing on the uses of the checkpoints not linked to the guidelines, in Figure 6 a great heterogeneity can be observed, and although it can be identified that the checkpoints of guidelines 2 and 1 are the least used, and those of guideline 5 are the most used. The rest are spread very thinly across the graph. At the same time, the fact that the most developed competences are identified implicitly, rather than explicitly, stands out.

## 4. Discussion

Based on the aforementioned results, and taking into consideration research questions 1–3, how are the different networks used?

### 4.1. Representation, action and expression, and engagement

With regard to the transmedia storytelling experiences collected for the systematic review, it can be stated that the *representation* network is quite underused, with 71.89% not developed. Within the transmedia storytelling experiences, it would be very positive to incorporate dynamics that take into consideration perception (G1) and language and symbols (G2).

Both guidelines are rarely used, but they are substantially different cases: In G2, we can see circumstances where it would be very complicated to give answers to the checkpoints and, again, it is not a question of always giving answers to these items, but rather of offering a teaching and learning proposal that contemplates the UD-L paradigm, not all the items are necessary for a good proposal in this framework. In the case of G2, there are few experiences linked, for example, to the field of mathematics, where it could make a lot of sense to work on checkpoint C2.1 and especially C2.3. On the contrary, in the case of G1, all the cases could develop it, and this is why it is a little more worrying not to identify it when many of the experiences either work on the literature or on the learning of a second language.

Although, in G1's interpretation, the technological aspect plays an important role: While in an analog context, the need for explicit adaptations would be clear, existing software can mediate the proposals without the need to make an intentional choice in the learning design, as such tools exist to facilitate access.

Regarding the *action and expression* network, it is the most used network, with a total of 59.47%. We believe that this figure could be even higher, but there are two items that decrease its use considerably.

The first*, optimize access to tools and assistive technologies*, a checkpoint where again—as in the case of G2—the question emerges as to what extent the incorporation of tools is taken for granted, does not exist, or simply does not emerge in the experiences collected. The second, C.5.3. *build fluencies with graduated levels of support for practice*, comes face to face with one of the aspects highlighted in the data retrieved for the study, given that the need for appropriate scaffolding for carrying out the activities was an element mentioned in several of the experiences as a need that perhaps they had not been able to resolve adequately or that needs to be taken into consideration. At the same time, and connecting with G4, G5, and their respective checkpoints, a bottleneck effect is identified: Many of the experiences are framed in a subject and context that does not allow for interdisciplinarity, whereas it would be a very natural element of participatory culture, and necessary to incorporate different mentoring figures for different approaches to the educational phenomenon.

Finally, as far as the *engagement* network is concerned, the ideas often linked to Jenkins' (2006) idea of participatory culture are generally very much incorporated, with the exception of C7.3 *minimize threats and distractions* and C8.4 *increase mastery-oriented feedback*. Again, C8.4 has certain points of contact with the issue of evaluation, as expressed in the case of C5.3 and C7.3.

One element that the case of C7.3 raises is that it is likely to presuppose that it is not necessary to establish mechanisms for distraction, given that one of the arguments for promoting transmedia narrative dynamics in the classroom is precisely this: to incorporate a narrative element that captures the students' attention, maximizing their motivation and interest. It should be added that many of the elements of engagement recovered in this section end up being collected thanks to the selection of tools such as blogs, forums, and other spaces for reflection, co-creation, and participation among peers.

### 4.2. Access, build, and internalize

If we look at it from the perspective of the *access, build*, and *internalize* categories, it is clear that the access perspective is underdeveloped. As noted earlier, there are three possible situations: (1) that it has been over-emphasized or omitted as a matter of space available to explain the experiences and research that emerges from it, (2) that digitization and tools that are often included within digital media are available to partially alleviate the accessibility problem, and (3) that it has not been considered.

In internalize, we find a fairly balanced use of the different checkpoints, and it is striking how in the build we find a great heterogeneity of uses between checkpoints. As noted earlier in reference to the least used checkpoints, this could be due to the type of experiences that are worked on within the SLR studies.

### 4.3. From a global perspective

**Figure 7:**
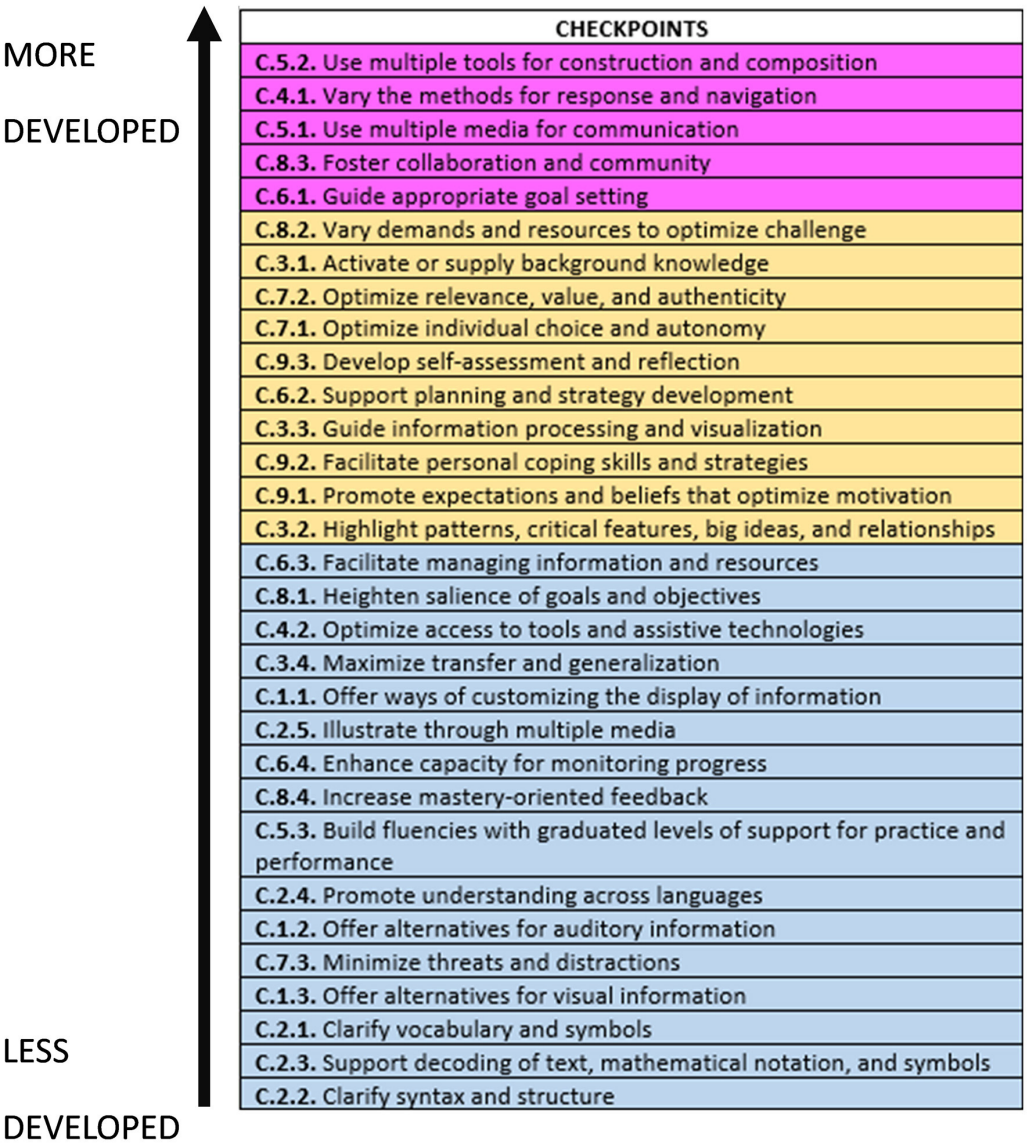
Checkpoints ranked according to their use from the highest to the lowest, and according to whether they are close to transmedia storytelling ideas (magenta), UD-L foundational research ideas (yellow), and accessibility (blue).

Considering the order from least to most that was previously visualized in Figure 6, it could be believed that, to a certain extent, the most used (marked in magenta in Figure 7) are those checkpoints more linked to participatory culture and to the ideas that Jenkins (2006) exposed. The aforementioned text has had a great impact on the literature and is one of the major references when talking about examples of transmedia narratives in the theoretical framework. It is, therefore, understandable that they include many of the elements of transmedia narrative expressed in their initial definition.

Following these checkpoints, others marked in yellow are identified, which are elements closely linked to the *foundational research* of the UD-L paradigm. According to the CAST (2018), these are elements rooted in aspects of neuroscience, cognitive psychology, and learning sciences.

Finally, the elements least observed in the experiences collected are those marked in blue and are related to the principle of accessibility and representation. During the SLR, we observed how the elements within the UD-L that seek to offer a multiplicity of modes of representation, monitoring, and assessment are the ones that are sometimes the most difficult to identify. These would be the ones that teachers should focus on when planning learning experiences, following the guidelines of CAST (2018) or Alba Pastor (2016), in order to better exploit the potential of transmedia storytelling from a UD-L and neuroscientific perspective.

### 4.4. Conclusion

In reference to the modes of representation, it is an element that is easy to integrate into a paradigm such as transmedia storytelling, where offering this multiplicity of means of representation is even an added value in the construction of the story, in working and unfolding the story through different media, and working in a crossmedia way with some elements, understanding that even working with the same content on another platform adds learning and ways of doing things that make it unique, since the channel partly defines the message (McLuhan, 1994; Dena, 2009). By the way of example, offering both the analog format of a book and its digital option, podcast, or online video explaining it, despite dealing with exactly the same content, the podcast and the physical book have very different characteristics between them, an element that we can take advantage of to generate greater engagement (especially in the autonomy of choice), but also to satisfy multiple modes, styles, and even learning rhythms.

Regarding monitoring and evaluation, it could be said that this is an element that needs to be worked on and requires more planning. In many of the activities, a more active and dynamic role in the teaching and learning processes would be positive, and simultaneously a process of co-construction among the students: Often the transmedia storytelling learning experiences are unidirectional: There is little peer evaluation and little collaboration among their projects. Small groups are proposed, allowing them to develop their competences and more active and full participation in the contents and competences to be deployed, but also more fragmented in their learning, and less connected both to the collective intelligence and to the reflections and learning of the rest of their peers.

An aspect that extends not only to the transmedia experiences identified but also to the difficulty of having sufficient information to replicate the transmedia narrative experiences analyzed, which do not offer enough information about these experiences to be able to participate in a real participatory culture where presuming, collaborating, or remixing around the existing experiences.

#### 4.4.1. Limitations of the study and future research

To conclude, transmedia storytelling as a didactic strategy still harbors a large number of possibilities to be discovered. But in order to be able to explore it, it is necessary to increase the transparency and transfer of these experiences, so that the experiences allow us to build meaningful experiences, and to avoid mistakes already made, and to suspect options, ideas, and try to guarantee shared educational actions that create a change of grammar and dynamics linked to educational processes that are closer to the daily realities of all the participants. The processes for which one is not always prepared, and, therefore, the need, as several of the studies described—and also the foundational aspects of UD-L—for a scaffolding for the activity, in line with not assuming that, by the mere fact of being digital natives, they necessarily have a high level of digital or transmedia literacy.

## Data availability statement

The raw data supporting the conclusions of this article will be made available by the authors, without undue reservation.

## Author contributions

RM-P: conceptualization, methodology, writing—original draft, writing—reviewing and editing, and visualization. JG-M: conceptualization, methodology, writing—original draft, and writing—reviewing and editing. All authors contributed to the article and approved the submitted version.
